# Robotic versus Laparoscopic Gastrectomy for Gastric Cancer: An Updated Systematic Review

**DOI:** 10.3390/medicina58060834

**Published:** 2022-06-20

**Authors:** Maurizio Zizzo, Magda Zanelli, Francesca Sanguedolce, Federica Torricelli, Andrea Morini, David Tumiati, Federica Mereu, Antonia Lavinia Zuliani, Andrea Palicelli, Stefano Ascani, Alessandro Giunta

**Affiliations:** 1 Surgical Oncology Unit, Azienda Unità Sanitaria Locale-IRCCS di Reggio Emilia, 42123 Reggio Emilia, Italy; andrea.morini@ausl.re.it (A.M.); david.tumiati@ausl.re.it (D.T.); federica.mereu@ausl.re.it (F.M.); antonialavinia.zuliani@ausl.re.it (A.L.Z.); alessandro.giunta@ausl.re.it (A.G.); 2 Pathology Unit, Azienda Unità Sanitaria Locale-IRCCS di Reggio Emilia, 42123 Reggio Emilia, Italy; magda.zanelli@ausl.re.it (M.Z.); andrea.palicelli@ausl.re.it (A.P.); 3 Pathology Unit, Azienda Ospedaliero-Universitaria, Ospedali Riuniti di Foggia, 71122 Foggia, Italy; francesca.sanguedolce@unifg.it; 4 Laboratory of Translational Research, Azienda Unità Sanitaria Locale-IRCCS di Reggio Emilia, 42123 Reggio Emilia, Italy; federica.torricelli@ausl.re.it; 5 Hematology Unit, CREO, Azienda Ospedaliera di Perugia, University of Perugia, 06129 Perugia, Italy; s.ascani@aospterni.it; 6 Pathology Unit, Azienda Ospedaliera S. Maria di Terni, University of Perugia, 05100 Terni, Italy

**Keywords:** gastric cancer, gastrectomy, robotic, laparoscopic, surgery, outcome

## Abstract

*Background and Objectives*: Gastrectomy with D2 lymphadenectomy is the standard surgical treatment with curative intent for patients with gastric cancer (GC). Over the last three decades, surgeons have been increasingly adopting laparoscopic surgery for GC, due to its better short-term outcomes. In particular, laparoscopic gastrectomy (LG) has been routinely used for early gastric cancer (EGC) treatment. However, LG suffers from technical limitations and drawbacks, such as a two-dimensional surgical field of view, limited movement of laparoscopic tools, unavoidable physiological tremors and discomfort for operating surgeon. Therefore, robotic surgery has been developed to address such limitations. *Materials and Methods*: We performed a systematic review following the Preferred Reporting Items for Systematic Reviews and Meta-Analyzes (PRISMA) guidelines in order to investigate the benefits and harms of robotic gastrectomy (RG) compared to the LG. PubMed/MEDLINE, Scopus, Cochrane Library (Cochrane Database of Systematic Re-views, Cochrane Central Register of Controlled Trials-CENTRAL) and Web of Science (Science and Social Science Citation Index) databases were used to search all related literature. *Results*: The 7 included meta-analyses covered an approximately 20 years-study period (2000–2020). Almost all studies included in the meta-analyses were retrospective ones and originated from Asian countries (China and Korea, in particular). Examined overall population ranged from 3176 to 17,712 patients. If compared to LG, RG showed both operative advantages (operative time, estimated blood loss, number of retrieved lymph nodes) and perioperative ones (time to first flatus, time to restart oral intake, length of hospitalization, overall complications, Clavien-Dindo (CD) ≥ III complications, pancreatic complications), in the absence of clear differences of oncological outcomes. However, costs of robotic approach appear significant. *Conclusions*: It is impossible to make strong recommendations, due to the statistical weakness of the included studies. Further randomized, possibly multicenter trials are strongly recommended, if we want to have our results confirmed.

## 1. Introduction

Being worldwide the fifth most common type of cancer and the fourth main reason of cancer mortality, gastric cancer (GC) has been recording the highest incidence and mortality rates in East and West Asia, Latin America and some East Europe countries [1,2].

Japanese, Korean, Chinese and Italian national guidelines recommend gastrectomy with D2 lymphadenectomy as standard surgical treatment with curative intent [3,4,5,6,7].

Over the last three decades, surgeons have been increasingly adopting laparoscopic surgery for GC, due to its better short-term outcomes [8,9,10,11]. In particular, laparoscopic gastrectomy (LG) has been routinely used for early gastric cancer (EGC) treatment [8,9,10,11]. Despite the strong interest, LG has been witnessing a rather rare use, due to poor evidence of long-term survival outcomes and concerns about oncological relevance of surgical resection [8,9,10,11]. Outcomes of robust trials proved the oncological safety of LG compared with open gastrectomy (OG) by showing non-inferiority in survival [8,9,10,11]. Moreover, technical problems related to total gastrectomy as well as D2 lymphadenectomy prevent the diffusion of laparoscopic surgery, as D2 lymphadenectomy entails the removal of node stations along celiac trunk, left gastric artery and hepatic pedicle [8]. Due to such reasons, execution of a correct D2 spleen-preserving LG in treating advanced gastric cancer (AGC) is reduced only in high-volume centers [8,9,10,11]. Difficult movement of laparoscopic tools, two-dimensional surgical field of view, unpreventable physiological tremors, in addition to surgeon’s discomfort in carrying out surgical procedures represent main LG drawbacks and limitations [8,9,10,11].

Robotic surgery aims at providing surgeons with high-resolution 3-D imaging, tools that ease wrist movement and a tremor filtering approach [8,9,10]. In 2003, Hashizume’s team reported robotic gastrectomy (RG) and since then number of patients undergoing such a procedure has been rapidly increasing [8].

Over the past 20 years, numerous primarily observational studies comparing outcomes by RG and LG for GC patients have been published [8]. In order to summarize the different data presented in the literature, quantify the effect and evaluate the heterogeneity of the included studies, several meta-analyses were presented, many of which collected in the synthetic (or “tertiary”) analysis/review (SAR) or umbrella review of Marano et al. in 2021 [8].

Starting from Marano et al.’s results [8], we carried out an updated systematic review by searching results of meta-analyses issued in the last two years, in order to provide surgeons with an update of the major scientific evidence about short- and long-term outcomes of the two minimally invasive approaches.

## 2. Materials and Methods

A systematic literature review was carried out by following Preferred Reporting Items for Systematic Reviews and Meta-Analyzes (PRISMA) statement and guidelines [12].

### 2.1. Search Strategy

Our search was carried out on PubMed/MEDLINE, Scopus, Cochrane Library (Cochrane Database of Systematic Reviews, Cochrane Central Register of Controlled Trials-CENTRAL) and Web of Science (Science and Social Science Citation Index). Combination of non-MeSH/MeSH terms was as follows:

- PubMed/MEDLINE

(((robotic(Title)) OR (robot(Title))) AND (gastrectomy(Title))) AND (gastric cancer(Title)) Filters: English, from 1 January 2020 to 31 January 2022.

- Scopus

(TITLE (robotic OR robot) AND TITLE ((gastrectomy) AND (gastric cancer) AND (LIMIT-TO (LANGUAGE, “English”) AND LIMIT-TO (DATE RANG, “2020–2022”)).

- Cochrane Library

Record Title OR robot in Record Title AND gastrectomy in Record Title AND gastric cancer in Record Title—with Cochrane Library publication date Between January 2020 and January 2022 (Word variations have been searched).

- Web of Science

(((TI = (robotic)) OR TI = (robot)) AND TI = (gastrectomy)) AND TI = (gastric cancer).

Refined by: LANGUAGES: (ENGLISH); PUBLICATION DATE: 1 January 2020 to 31 January 2022.

Final analysis was carried out in February 2021.

### 2.2. Inclusion Criteria

Just English-language scientific articles published from 1 January 2020 to 31 January 2022 were taken into account.

Our systematic review covered meta-analyses of studies comparing adult patients (over 18 years of age) who underwent robotic or laparoscopic gastrectomy (Robotic Gastrectomy RG—versus Laparoscopic Gastrectomy LG) for GC.

In order to further identify possibly relevant articles, we resorted to a manual scanning of reference lists for the included studies.

### 2.3. Data Extraction

The papers were selected and identified by two independent reviewers (M.Zi. and A.G.) based on title, abstracts, keywords and full-texts. The following information was collected from selected papers:

- demographic data: author’s surname and year of publication, database used, included studies [number, type, period, country, language], surgical procedure, population size;
- meta-analyses data: operative outcomes [operative time, estimated blood loss, conversion to open surgery, number of retrieved lymph nodes, length of proximal and distal resection margins]; perioperative outcomes [time to first flatus, time to restart oral intake, length of hospitalization, overall complications, Clavien-Dindo (CD) ≥ III complications, mortality, reoperation, anastomotic leakage, pancreatic complications, delayed gastric emptying, intestinal obstruction, wound infection, intra-abdominal infection, duodenal stump leakage, anastomotic stenosis, abdominal bleeding, ileus, pneumonia]; long-term outcomes [overall survival, recurrence-free survival, recurrences, costs]. Sample size, heterogeneity, overall effect size, 95% of overall effect, *p*-value were included into the study number.

In the end, a third independent reviewer (M.Za.) analyzed all collected results.

### 2.4. Quality Assessment

The quality of included studies was independently assessed by two reviewers (M.Zi and M.Za.) by use of A Measurement Tool to Assess Systematic Reviews—2 (AMSTAR-2) [13]. Following guidelines and taking into account 16 possible critical and noncritical weaknesses, 4 global quality ratings (high, moderate, low or critically low) were set up [13]. Items addressing following criteria (clear research question, including definitions of population, intervention, control group and outcomes, adequacy of literature search and proper assessment and/or consideration of risk of bias in the primary studies) were considered as critical ones [13]. High and moderate ratings mark one or different not-serious defects, while low and very low ratings indicate one or different serious weaknesses [13]. Any inconsistency among independent raters was solved through discussion [13].

## 3. Results

### 3.1. Search Results and Study Characteristics

**Figure 1:**
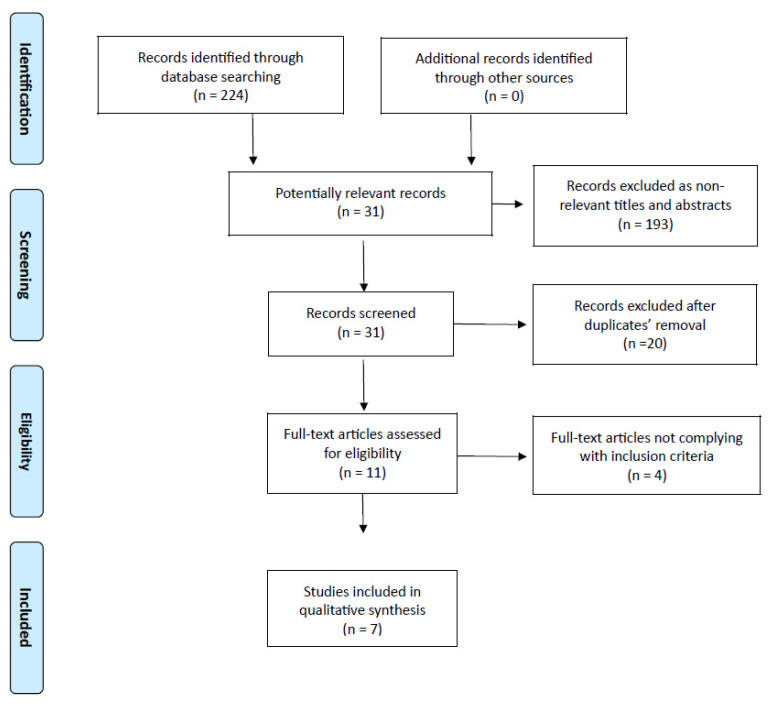
PRISMA flow chart of literature search.

Final literature search performed on 31 January 2022 identified 224 studies of potential interest (Figure 1). Analysis involved all 224 studies: out of them, 193 ones turned out as not relevant for title and abstract, while 31 full-texts were considered eligible. Twenty of the 31 studies were excluded because they were identified as duplicate publications. After removal of 4 studies not complying with inclusion criteria (1 robotic gastrectomy versus open gastrectomy meta-analysis; 1 umbrella review; 1 umbrella review protocol; 1 network meta-analysis), 7 meta-analyses underwent qualitative synthesis [14,15,16,17,18,19,20]. No additional record was found through other source (References list). According to the AMSTAR-2 tool, we identified 5 low quality and 2 high quality studies.

### 3.2. General Population Characteristics

Table 1 shows demographic features of 7 included meta-analyses that covered 2000–2020 time frame [14,15,16,17,18,19,20]. Studies included into the meta-analyses ranged from 12 to 40, were almost all retrospective ones and originated from Asian countries (China and Korea, in particular) [14,15,16,17,18,19,20]. Examined overall population ranged from 3176 to 17,712 patients; in all studies, the laparoscopic subgroup turned out as more represented than the robotic one [14,15,16,17,18,19,20]. Almost all researches detailed surgical adopted procedure, with a prevalence of distal and total gastrectomy [14,15,16,17,18,19,20].

### 3.3. Operative Outcomes

**Figure 2:**
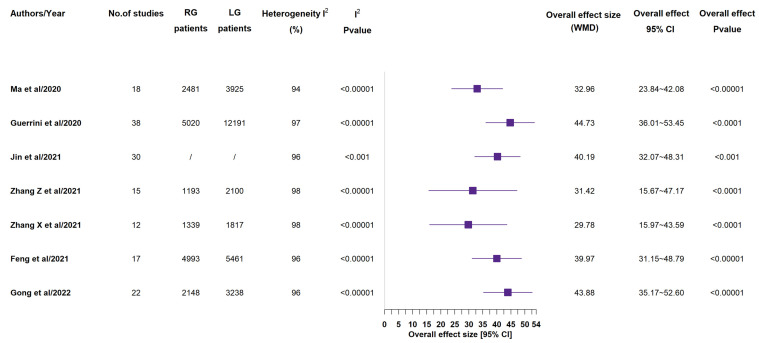
Operative time (min). RG = Robotic gastrectomy; LG = Laparoscopic gastrectomy; WMD = weighted mean difference [14,15,16,17,18,19,20].

**Figure 3:**
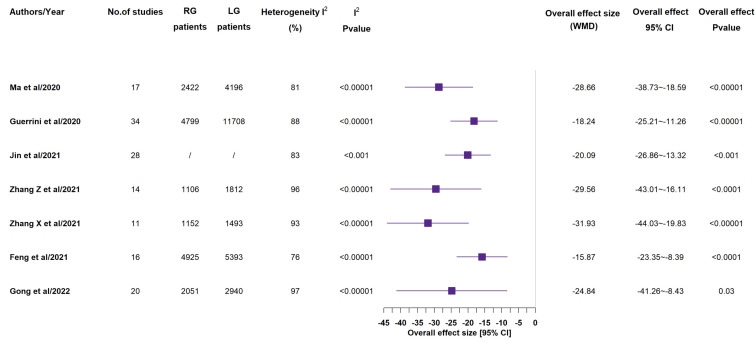
Estimated blood loss (mL). RG = Robotic gastrectomy; LG = Laparoscopic gastrectomy; WMD = weighted mean difference [14,15,16,17,18,19,20].

According to all included meta-analyses, operative time (Figure 2) was longer in RG group than in LG one [12,13,14,15,16,17,18]. In all 7 studies, this result had statistical significance [14,15,16,17,18,19,20]. All included meta-analyses reported lower estimated blood loss (Figure 3) in RG group than in LG one: also in this case, all 7 studies gave statistical significance to such a result [14,15,16,17,18,19,20]. Just 3 studies out of the 7 ones reported that rate of conversion to open surgery (see Supplementary Materials-Table S1) showed no statistically significant difference between the two groups [15,16,19].

**Figure 4:**
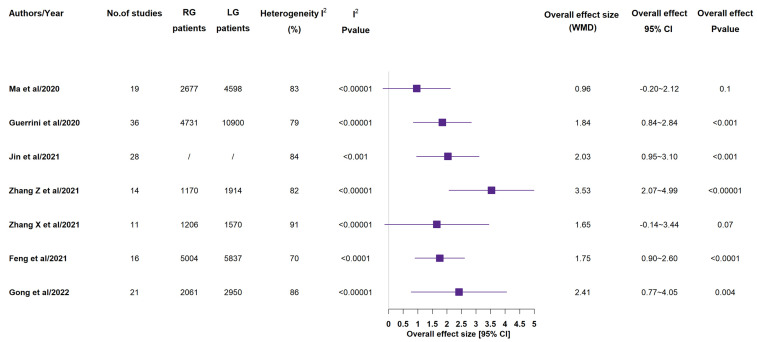
Retrieved lymph nodes (n). RG = Robotic gastrectomy; LG = Laparoscopic gastrectomy; WMD = weighted mean difference [14,15,16,17,18,19,20].

Taking into account histological parameters, all included meta-analyses recorded the number of retrieved lymph nodes (Figure 4) [14,15,16,17,18,19,20]. In 5 studies out of the 7 ones, RG group showed a statistically significant higher number of retrieved lymph nodes, in comparison to LG group [15,16,17,19,20]. With the exception of Zhang X et al.’s paper, meta-analyses reported length of proximal and distal resection margins [14,15,16,17,19,20]. In none of the included studies, the proximal resection margin length (see Supplementary Materials-Table S2) showed a statistically significant difference between the two groups, while 2 out of the 6 included studies recorded a statistical more significant distal resection margin length (see Supplementary Materials-Table S3) in the RG group in Jin et al.’s study and in the LG group in Zhang Z et al.’s study [12,13,14,15,17,18].

### 3.4. Perioperative Outcomes

**Figure 5:**
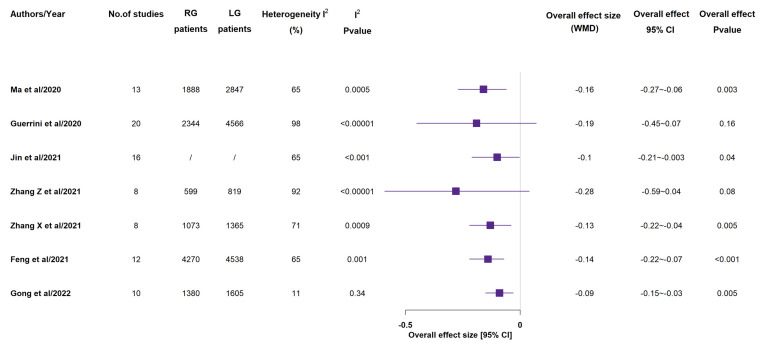
Time to first flatus (days). RG = Robotic gastrectomy; LG = Laparoscopic gastrectomy; WMD = weighted mean difference [14,15,16,17,18,19,20].

**Figure 6:**
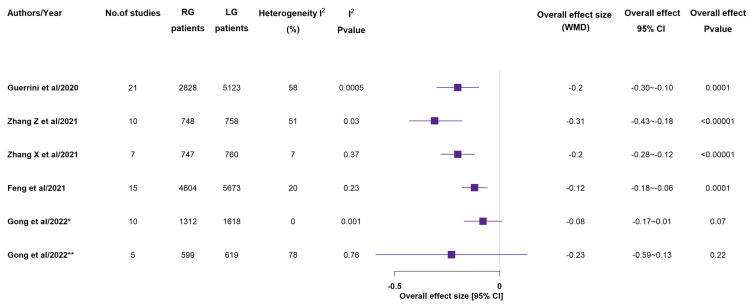
Time to restart oral intake (days). RG = Robotic gastrectomy; LG = Laparoscopic gastrectomy; WMD = weighted mean difference; * liquid diet; ** solid soft diet [15,17,18,19,20].

**Figure 7:**
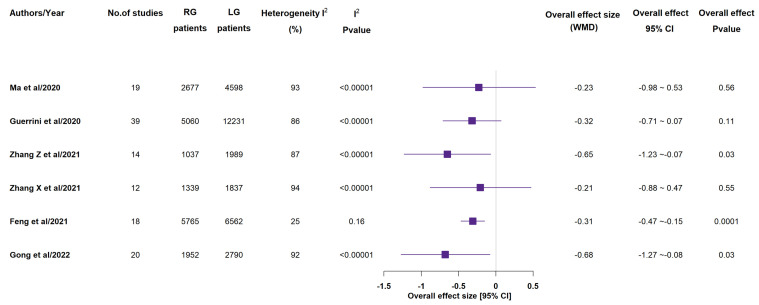
Length of hospital stay (days). RG = Robotic gastrectomy; LG = Laparoscopic gastrectomy; WMD = weighted mean difference [14,15,17,18,19,20].

All included meta-analyses reported time to first flatus (Figure 5) and 4 of them recorded a statistically shorter time in RG group than in LG one [14,15,16,17,18,19,20]. Moreover, 4 meta-analyses out of the 5 ones, that reported time to restart oral intake (Figure 6), detected a statistically significant shorter time in RG group than in LG one [15,17,18,19,20]. With the exception Jin et al.’s paper, 6 meta-analyses reported length of hospitalization (Figure 7) [14,15,17,18,19,20]. 3 out of 6 studies showed statistically significant shorter length of hospitalization in RG group than in LG one [17,19,20].

**Figure 8:**
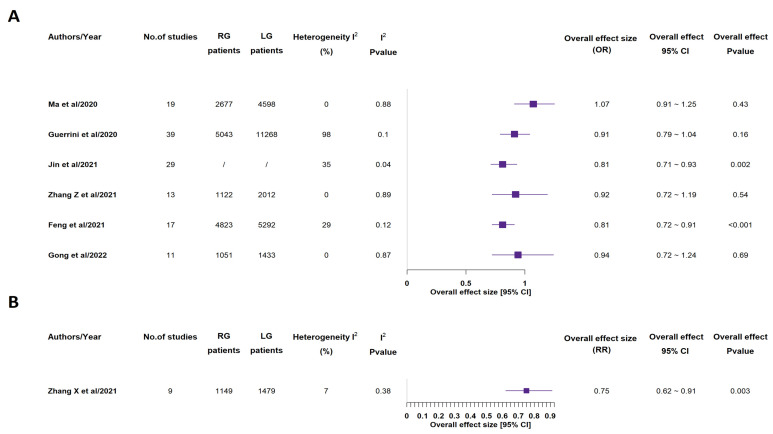
Overall complications rate. RG = Robotic gastrectomy; LG = Laparoscopic gastrectomy; OR = odds ratio (**A**); RR = risk ratio (**B**) [14,15,16,17,18,19,20].

All included meta-analyses recorded overall complications (Figure 8) and mortality rates (see Supplementary Materials-Table S4) [14,15,16,17,18,19,20]. 3 out of the 7 studies found a statistically significant lower overall complication rate in RG group, while all studies did not find any significant discrepancy between the two groups, in terms of mortality rate [14,15,16,17,18,19,20].

**Figure 9:**
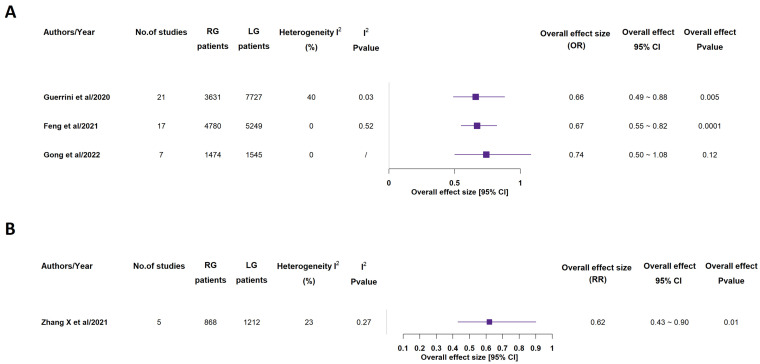
CD > III complications rate. RG = Robotic gastrectomy; LG = Laparoscopic gastrectomy; OR = odds ratio (**A**); RR = risk ratio (**B**) [15,18,19,20].

**Figure 10:**
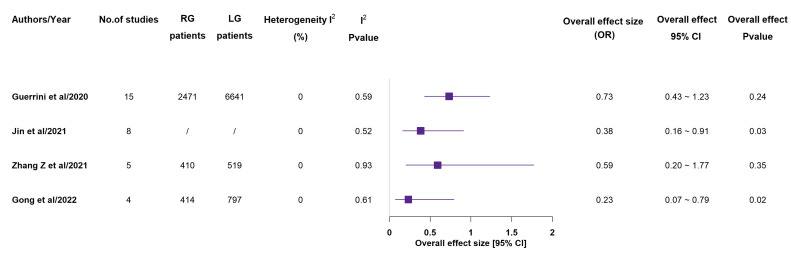
Pancreatic complications rate. RG = Robotic gastrectomy; LG = Laparoscopic gastrectomy; OR = odds ratio [15,16,17,20].

Four out of seven included meta-analyses reported CD ≥ III complication rate (Figure 9) and according to 3 of them, RG group highlighted a statistically significant lower rate, when compared to LG group [15,18,19,20]. Many other specific issues were reported by one or more included meta-analyses. As concerned pancreatic complication rate (Figure 10), 2 studies out of the 4 reporting ones detected a statistically significant lower rate in the RG group, although no statistically significant difference between the groups was found as concerned reoperation, anastomotic leakage, delayed gastric emptying, intestinal obstruction, wound infection, intra-abdominal infection, duodenal stump leakage, anastomotic stenosis, abdominal bleeding, ileus, pneumonia rates (see Supplementary Materials-Tables S5–S15) [14,15,16,17,18,19,20].

### 3.5. Long-Term Outcomes and Costs

Out of the 7 included studies, 3 ones recorded overall survival, 2 ones reported recurrence-free survival and 3 ones detected recurrence rates (see Supplementary Materials-Tables S16–S18), while no statistically significant differences between the two groups were recorded [14,15,16,17,19].

**Figure 11:**
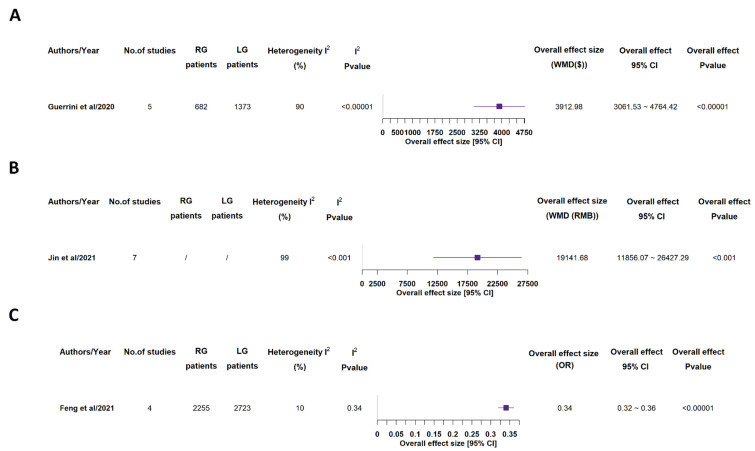
Costs. RG = Robotic gastrectomy; LG = Laparoscopic gastrectomy; WMD = weighted mean difference; $ = US dollars (**A**); RMB = People’s Republic of China renminbi (**B**); OR = odds ratio (**C**) [15,16,19].

Costs (Figure 11) were analyzed in just 3 studies out of 7 ones, and in all cases they turned out as statistically significant higher in RG group than in LG one [15,16,17,19].

## 4. Discussion

Development of robotic equipment and accumulation of surgical experience following Hashizume et al. first RG performance in 2003 made RG an increasingly followed approach in GC, which now represents a large-scale surgical procedure [8]. At present, an increasing number of studies has been exploring RG safety and effectiveness in GC treatment [8]. Several studies proved that GC treatment through RG is both safe and feasible, while its healing consequences are similar to those of OG, although it requires higher surgical skills and longer times [8,21]. However, RG safety and efficacy in comparison to LG ones are strongly debated in the treatment of GC.

Starting from 2021 Marano et al.’s results dealing with a comparison of outcomes by robotic surgery and laparoscopic one for GC, we carried out an updated systematic review, just including meta-analyses issued from January 2020 to January 2022 [8]. In particular, we identified 7 new meta-analyses on the afore mentioned topic and no one was included in Marano et al.’s umbrella review [14,15,16,17,18,19,20]. Discussion of results must be introduced by possible biases that should not be ignored. The included meta-analyses were almost all retrospective studies that referred to very heterogeneous populations, mainly stemming from Eastern countries, thus explaining the low quality of 5 out of 7 meta-analyses (according to AMSTAR-2 grade). Furthermore, the presence of comparable results among the meta-analyses that we identified may be related to the inclusion of the same studies (duplicate publication bias), coming from the Eastern countries and referred to 2014–2018 time frame of publication.

All included meta-analyses highlighted a statistically significant longer operative time in the robotic group than in the laparoscopic one [14,15,16,17,18,19,20]. On one hand, the reason could be found in the setting up and docking of robotic arms, which required longer operating times [14,15,16,17,18,19,20]. Studies showed an approximately 30-min setting up for robotic surgery [14,15,16,17,18,19,20]. On the other hand, different experiences by surgeons could explain longer operative times [14,15,16,17,18,19,20]. First, docking times can be reduced by more experienced surgeons [14,15,16,17,18,19,20]. Moreover, RG learning curve can increase operating time [14,15,16,17,18,19,20]. By developing Da Vinci robotic surgery system, greater experience and a shorter learning curve can make robotic surgery more effective and operating times shorter [14,15,16,17,18,19,20].

In addition to operative time, estimated blood loss represents one of the major concerns for surgeons as quality indicator. As it happened with operative times, all 7 included studies reported a statistically significant result by robotic group, that recorded a lower estimated blood loss if compared to laparoscopic one [14,15,16,17,18,19,20]. This result can be explained by robotic surgical system’s inherent advantages over laparoscopy [14,15,16,17,18,19,20]. RG allows a three-dimensional 10-to-15 time magnified operating field, thus helping surgeons to have a more direct and clear view of blood vessels and surrounding tissues, in addition to recognizing tissual structure [14,15,16,17,18,19,20]. Moreover, robotic “hands” may be useful, as they lack unintentional shaking which is typical of human hands [14,15,16,17,18,19,20]. In addition to improving intervention durability and accuracy, such method allows dissection safety and ligation of gastric blood vessels [14,15,16,17,18,19,20].

Number of retrieved lymph nodes is one of the most significant indicators of radical gastrectomy effectiveness [3,4,5,6]. Extensive dissection of an adequate number of lymph nodes does not only improve the accuracy in staging patient’s lymph node metastases, but also reduces risk of recurrence and metastasis for the patient [3,4,5,6]. All 7 included meta-analyses reported this parameter and 5 out of them identified a statistically significant greater number of lymph nodes retrieved in the RG group, in comparison to the LG one [14,15,16,17,18,19,20]. Among those 5 studies, we found that of Guerrini et al., which included the largest number of metanalysed observational studies [15]. As it happened in the case of estimated blood loss, the main reason for the above result was that RG has three-dimensional imaging, a tremor filter and an internal articulated EndoWrist with 7 degrees of freedom, and all this contributed to more precise dissection and lymphadenectomy, especially for the lymph nodes of soft tissue surrounding gastric vessels [14,15,16,17,18,19,20]. Moreover, it might also be related to the continuous advancement of the robotic surgery system and the improvement of surgeons’ proficiency in its operation [14,15,16,17,18,19,20].

Length of proximal and distal resection margins represent other oncological safety parameters. 6 out of the 7 meta-analyses reported this information: 2 out of them showed a weak but statistically significant greater length of distal resection margin in the RG group in Jin et al.’s study and in the LG group in Zhang Z et al.’s study, in the absence of great differences in all studies between the two groups, as concerned length of proximal resection margin [14,15,16,17,19,20]. Therefore, the two minimally invasive approaches may be useful in obtaining good proximal and distal resection margins.

Taking into account perioperative outcomes, we found that RG group showed statistically significant shorter time to first flatus (4 studies out of 7 ones) and shorter time to first oral intake (3 studies out of 4 ones) if compared to LG one [14,15,16,17,18,19,20]. According to Ma et al., those results might be associated to stable and flexible movements of robotic arms, by avoiding excessive traction on the tissue, accidental injury to blood vessels, and by reducing trauma to patients [14]. However, we identified a statistically significant shorter length of hospital stay for the RG group in just 3 out of 6 studies, and we cannot rule out that this arises from possible discrepancies among studies, according to application or disapplication of Enhanced Recovery After Surgery (ERAS) protocols [14,15,17,18,19,20].

Furthermore, RG group recorded statistically significant lower overall complication rate (3 out of 7 studies) and lower CD ≥ III complication rate (3 out of 4 studies), if compared to LG one [14,15,16,17,18,19,20]. However, with the only exception of pancreatic complication rate (2 out of 4 studies), no statistically significant differences were identified between the two groups, as concerned rates of reoperation, anastomotic leakage, delayed gastric emptying, intestinal obstruction, wound infection, intra-abdominal infection, duodenal stump leakage, anastomotic stenosis, abdominal bleeding, ileus, and pneumonia [14,15,16,17,18,19,20].

Finally, despite statistically significant higher number of retrieved lymph nodes in the RG group compared to the LG one, we found no difference between the two groups, in terms of overall survival, recurrence-free survival and recurrence rates [14,15,16,17,19]. This last result seems to be significant, as it underlines oncological efficacy of both minivasive methods, although costs related to robotic gastric surgery are significantly higher [15,16,17,19].

The above-mentioned meta-analyzed results could have been more or less affected by many factors. Among them, the learning curve, new imaging approaches (e.g., Indocyanine green (ICG) fluorescence imaging) and distinction between robotic systems (e.g., da Vinci Si^®^ (Intuitive Surgical Inc., Sunnyvale, CA, USA) versus da Vinci Xi^®^ (Intuitive Surgical Inc., Sunnyvale, CA, USA)) arouse remarkable surgical interest. Therefore, it is paramount to discuss the above and in general about availability and costs related to robotic surgery.

In laparoscopic distal and total gastrectomies, estimated learning curve may record proficiency levels of 60–90 and 100 cases, respectively [22,23,24]. Although, no comparative head-to-head studies have been carried out so far, the learning curve in robotic distal and total gastrectomies is assumed to be lower than in laparoscopic ones [22]. Technical benefits provided by robotic system in addition to considerable ergonomic improvements can justify this assumption [22]. This applies especially in case of skilled surgeons in laparoscopic gastric surgery [22]. In 2009 Song et al. assessed the impact of surgeons’ experience on perioperative outcomes following minimally invasive distal gastrectomy; they compared three groups of surgeons after dividing their skills into early laparoscopic (EL) and late laparoscopic (LL) and early robotic (ER) (three groups of 20 cases each) [25]. They found that LL and ER groups recorded corresponding outcomes, although they were better in comparison to EL group [25]. These results were validated by Park et al.’s study, that highlighted the significant impact of laparoscopic experience on shorter operative times in robotic distal gastrectomy [26]. Therefore, Vining et al. came to the conclusion that well experienced surgeons in laparoscopic gastric surgery recorded a learning curve of RG of about 11–25 cases, while skilled surgeons without laparoscopic experience recorded a learning curve of 20–25 cases [22]. Training programs in robotic surgery could lead to important consequences on learning curve, but no studies on this subject are available at the moment [22]. A prospective multicenter study by Kim et al. has recently analyzed the morbidity related to learning curve following RG [27]. Analyzing the performances of 5 skilled surgeons (more than 60 robotic gastrectomies), the study led to following results: an initial proficiency in operating times (following approximately 25 cases); minimum operating time and a plateau (following approximately 65 cases) [27]. Moreover, as far as CD ≥ 2 complications are concerned, after an initial proficiency phase (26–65 cases), a rebound increase of complication rates (66–88 cases) was recorded, while the lowest complication rate was recorded after 88 cases in a row [27]. The rebound phase was both related to extension of indications and increased attempts to perform more demanding technical procedures [27]. However, it must be taken into account that many factors may affect the learning curve: type of console, docking time, type of gastric resection and lymph node dissection, previous experience in open and/or laparoscopic gastric surgery [27]. Almost all meta-analyses that we took into account defined the learning curve as one key factor in the quality of the gained results [14,15,16,17,18,19,20]. However, no study has carefully analyzed the statistical significance of this impact (long-term oncological outcomes, in particular) [14,15,16,17,18,19,20].

Indocyanine green (ICG) fluorescence imaging represents one of the most recent widespread innovations in minimally invasive surgery, that can be employed in both robotic and laparoscopic surgery. High-quality minimally invasive D2 lymphadenectomy requires considerable technical expertise and may not be easily performed everywhere [28]. In particular, an optimal lymph node dissection (LND) near to the great vessels may cause unexpected iatrogenic damage, especially among surgeons under training or less skilled ones; this may lead the latter to reduce the risk by performing a lower quality LND, with a potential impact on long-term prognosis. However, ICG fluorescence imaging in radical gastrectomy remains a controversial issue, due to the lack of high-quality evidence. Two recent meta-analyses showed that ICG fluorescence imaging led to a higher number of harvested lymph nodes, lower intraoperative blood loss, and shorter hospitalization length, if compared to conventional surgery [28,29]. As a matter of fact, following ICG administration, a clearer identification of the lymphatic structures takes place and fluorescent lymph nodes seem more easily recoverable, leaving the neighboring structures (large vessels, in particular) unaltered, although Yang et al. did not find any advantage in terms of overall and specific postoperative complications in the ICG group [28,29]. Our paper records an advantage of RG over LG, as far as number of recovered lymph nodes and estimated intraoperative blood loss are concerned [14,15,16,17,18,19,20]. However, included meta-analyses did not identify the impact of ICG fluorescence imaging, as almost all included studies did not make use of visual tools [14,15,16,17,18,19,20]. Therefore, it cannot be ruled out that the use of ICG fluorescence imaging in LG may lead to a reduced statistical significance up to the missing of significant discrepancies between RG and LG, both in terms of recovered lymph nodes and intraoperative blood loss.

Nowadays, the da Vinci system (Intuitive Surgical Inc., Sunnyvale, CA, USA) represents the reference robotic platform at world level. In the last 20 years, various versions of the platform marked by useful and significant updates have entered the market. The da Vinci Xi^®^ system, that represents the latest robotic surgery system, was introduced to overcome previous platform’s (da Vinci Si^®^ system) limitations [30,31]. Due to its overhead arm rotation without axis limitation and thanks to its laser targeting device that allows for optimal arm positioning, it provides improved anatomical access for multi-quadrant surgical procedures [30,31,32]. In addition, it is equipped with an 8-mm endoscope that requires neither draping, nor autofocus or white balance and can be used with all arms [30,31,33]. Finally, the da Vinci Xi^®^ system has narrower arms and a longer instrument shaft, which offers the surgeon a better reach [30,31,34]. Either use of one version or the other may have had an impact on above described results. However, no meta-analyses focused on this issue [14,15,16,17,18,19,20]. To date, just two retrospective studies compared different versions of the robotic platform in gastric surgery [30,31]. Comparing da Vinci Xi^®^ (107 patients) with da Vinci Si^®^ (179 patients) in RG for GC at their Institution, Alhossaini et al. proved that both groups had similar operative (total operative time, docking time, console time, estimated blood loss, conversion rate), pathological (number of retrieved lymph nodes) and perioperative (days to first flatus, days to sips of water, days to liquid diet, day to first soft diet, complications rate, complications grade rate, hospital length of stay) outcomes [30]. Two years later (2021), Ojima et al. extended the comparison to the Da Vinci S^®^ system (Intuitive Surgical Inc., Sunnyvale, CA, USA) (20 patients; Si^®^ 30 patients, Xi^®^ 98 patients) [31]. Authors recorded significantly longer total operative times and console times in group S^®^, while docking times turned out significantly longer in group Xi^®^ [31]. Authors attributed the aforementioned differences to 2 factors: technological inferiority of S^®^ system, if compared to other two ones and developing learning curves of surgeons [31]. However, no other parameters taken into account between the three groups seemed significantly different [31].

Unlike what happened up to 10 years ago, when it was struggling to spread out, robotic surgery has been turning into an integral part of clinical practice, due to different advantages of performing increasingly complex dissections and reconstructions in different surgical fields, when compared to traditional laparoscopic surgery [9,22,35,36,37]. It is especially true in gastric surgery, whose minimally invasive reconstructions during total gastrectomy seem more challenging (esophagojejunal anastomosis, in particular), if compared to those performed during partial gastrectomies. To our great surprise, ruling out 2 of 7 included meta-analyses, that investigated only distal gastrectomy [17,20], just Jin et al.’s study performed a subgroup analysis by type of gastric resection [16]. In terms of distal gastrectomy, if compared to LG group, authors highlighted that RG group showed longer operative time, lower estimated blood loss and higher number of lymph nodes retrieved [16]. Furthermore, time to first flatus and hospitalization length were comparable between the two procedures [16]. As concerned total gastrectomy, they indicated that RG required longer operative time but had a much shorter time to first flatus, while estimated blood loss, hospitalization length, and number of lymph nodes retrieved were similar between the two procedures [16]. In the last few decades, several studies on the outcomes of laparoscopic distal gastrectomy (particularly for patients affected by early gastric cancer) have been issued [8,9,10,11] The studies on the outcomes of laparoscopic total gastrectomy are significantly lower due to the aforementioned surgical technical difficulties [8,9,10,11]. Therefore, the contribution of robotic surgery seems to be of paramount importance. Recently, two prospective pioneer studies on robotic and laparoscopic total radical gastrectomy for advanced gastric cancer have been issued, whose results showed that robotic total gastrectomy bears the advantage of less intraoperative bleeding, faster postoperative recovery with less trauma and more lymph nodes recovered [38,39]. Unlike Lin et al., who did not record any significant differences, Chen et al. pointed out a longer operative time and a lower rate of major complications in the robotic total gastrectomy group [38,39].

The now consolidated laparoscopic expertise of III level gastrointestinal surgeons led those Centers and Health Institutions in general to examine robotic surgery thanks to an increasing availability of robotic platforms. This is especially true in Western Centers, where the spreading of robotic surgery has taken place at a slower pace, when compared to Asian Centers. Emilia-Romagna, the Northern Italian Region where our hospital is located, went from a 2005-installed platform (Civil Hospital of Baggiovara, Modena, Italy) to 5 further robotic platforms, that were introduced between 2014 and 2021 [40].

Nowadays, commercially available robotic equipment is marked by high costs, including acquisition costs, training expenses and costs of equipment-tool, in addition to maintaining costs for robotic system [41]. So high costs may justify the slowing spread of robotic assisted surgery [9,22,35,36,37,41,42]. According to van Dam et al., in order to offset the initial costs for the acquisition of robotic devices, robotic surgery should be employed in more than 300 interventions per year, for 7 years, totalling up an amount of over 1000 euros per patient [43]. As a consequence, it can be easily explained why robotic surgery exclusively refers to Health Centers with a large volume of patients, in order to get the lowest possible per-case rates [43]. In the present financial situation, when most world countries find it difficult to support existing healthcare facilities, a new and very expensive technique such as robotics would hardly take hold as a new standard of care, in the lack of clear evidence of significant cost-effectiveness by comparing minimally invasive methods and standard surgical techniques [41]. Many factors could impact on costs: operating time, skills in the surgical team and learning curves of surgeons, length of hospitalization, type of surgery, etc.

Training of specialized robotic units yearly operating on a large number of cases, reduction of operative time through specialized training on robotic approach, early discharge of patients whenever it is possible and a reduced number of tools used per each intervention represent the first steps towards cost reduction [41]. Multiple use of robotic equipment by multiple surgical specialties, good training of all team members involved could represent a useful suggestion to decrease costs, while robotic devices could be purchased through research funding or even charities [41].

Summing up the outcomes of our Results and Discussion sections, it turned out that, at present, robotic gastric surgery can be performed just by highly experienced centers. No robust results can justify the choice of robotic gastric surgery, as laparoscopic gastric surgery still leads to the best cost-benefit balance.

### Limitations

Our systematic review bear some limitations: (i) literature search did not include non-English-written scientific papers; (ii) included studies were almost exclusively non-randomized retrospective series; (iii) populations under analysis showed significant heterogeneity; (iv) gastric cancer stage was not specified in many of the included studies; (v) the majority of the studies were from Eastern populations; (vi) some of the population studies were present in two or more of the 7 meta-analyses included (duplicate publication bias). For all those reasons direct comparison of findings turned out difficult.

## 5. Conclusions

To sum on up, our updated systematic review, that included recently published meta-analyses, allowed us to confirm what scientific literature had already reported.

In particular, if compared to LG, RG showed both operative advantages (operative time, estimated blood loss, number of retrieved lymph nodes) and perioperative ones (time to first flatus, time to restart oral intake, length of hospitalization, overall complications, CD ≥ III complications, pancreatic complications), in the absence of clear discrepancies of oncological outcomes. However, costs of robotic approach appear significant.

Regardless of the above results, it is impossible to make strong recommendations, due to the statistical weakness of the included studies (retrospective studies and high heterogeneity, in particular). Further randomized, possibly multicenter trials may turn out as very precious in confirming our results.

## Figures and Tables

**Table 1:** General population characteristics.

Authors/Year	Database	Studies Included												Surgical Extension					Patient Population			AMSTAR-2
		Number	Type			Period	Country						Language	P	D	T	PPG	Not Described	Overall	RG	LG	
			Restrospective	Prospective	RCT		China	Japan	Korea	Taiwan	Italy	USA										
Ma et al./2020 [14]	Pubmed, Cochrane Library, WanFang, CNKI, VIP	19	19	0	0	2003–2019	13	2	4	0	0	0	English, Chinese	7	17	14	1	0	7275	2677	4598	Low
Guerrini et al./2020 [15]	Pubmed, MEDLINE, Cochrane Library	40	11	29	0	2003–2019	8	7	21	1	3	0	English	5	36	28	2	0	17,712	5402	12,310	Low
Jin et al./2021 [16]	Pubmed, Embase, Cochrane Library	31	30	0	1	/	13	3	12	0	2	1	English	/	6	3	/	22	12,401	4274	8127	Low
Zhang Z et al./2021 [17]	Pubmed, Embase, Cochrane Library, Web of Science	15	15	0	0	2003–2018	9	1	5	0	1	0	English, Chinese	0	15	0	0	0	3293	1193	2100	Low
Zhang X et al./2021 [18]	Pubmed, Embase Cochrane Library, WanFang, CNKI, VIP	12	12	0	0	2000–2019	10	0	1	0	1	0	All	1	9	4	/	2	3176	1339	1837	Low
Feng et al./2021 [19]	Pubmed, Embase, Cochrane Library, Web of Science	20	19		1	2005–2020	11	2	5	0	1	1	English	/	/	/	/	20	13,446	6173	7273	High
Gong et al./2022 [20]	Pubmed, Embase, Cochrane Library, Web of Science	22	22	0	0	2000–2020	5	5	9	0	3	0	English	0	22	0	0	0	5386	2148	3238	High

AMSTAR = A MeaSurement Tool to Assess systematic Reviews; P = Proximal gastrectomy; D = Distal gastrectomy; T = Total gastrectomy; PPG = Pylorus-preserving gastrectomy; RCT = Randomized control trial; RG = Robotic gastrectomy; LG = Laparoscopic gastrectomy.

## Data Availability

The data presented in this study are available on request from the corresponding author.

## Supplementary Materials

The following supporting information can be downloaded at: https://www.mdpi.com/article/10.3390/medicina58060834/s1, Table S1: Conversion to open surgery; Table S2: Proximal margin length; Table S3: Distal margin length; Table S4: Mortality; Table S5: Reoperation rate; Table S6: Anastomotic leakage; Table S7: Delayed gastric emptying; Table S8: Intestinal obstruction; Table S9: Wound infection; Table S10: Intra-abdominal infection; Table S11: Duodenal stump leakage; Table S12: Anastomotic stenosis; Table S13: Abdominal bleeding; Table S14: Ileus; Table S15: Pneumonia; Table S16: Overall survival; Table S17: Recurrence free-survival; Table S18: Recurrence.
