# Supplementary material for: Robotic versus Laparoscopic Gastrectomy for Gastric Cancer: An Updated Systematic Review
Source: Medicina (Kaunas). 2022 Jun 20;58(6):834. doi: 10.3390/medicina58060834 (PMC9231199; doi:10.3390/medicina58060834)
Supplement: Supplementary file 1 [file medicina-58-00834-s001.zip › medicina-1753701-supplementary.pdf]

Supplementary material – Other outcome parameters of interest

RG = Robotic gastrectomy; LG = Laparoscopic gastrectomy

Tables S1. Conversion to open surgery.

**Authors/Year**	**No. of studies**	**Sample size**		**Heterogeneity**		**Overall effect size**	**95% CI of overall effect**	**P value**
		**RG**	**LG**	**I^2 (%)**	**P value**			
Guerrini et al/2020 [15]	28	3777	9584	1	0.43	OR = 0.76	0.45 ~ 1.28	0.30
Jin et al/2021 [16]	7	/	/	0	0.65	OR = 0.86	0.44 ~ 1.66	0.65
Feng et al/2021 [19]	4	2301	2567	0	0.62	OR = 0.62	0.40 ~ 1.07	0.09

Tables S2. Proximal margin length.

**Authors/Year**	**No. of studies**	**Sample size**		**Heterogeneity**		**Overall effect size**	**95% CI of overall effect**	**P value**
		**RG**	**LG**	**I^2 (%)**	**P value**			
Ma et al/2020 [14]	7	761	1252	28	0.22	WMD = 0.10	- 0.09 ~ 0.29	0.30
Guerrini et al/2020 [15]	13	1940	4978	23	0.21	WMD = 0.01	- 0.14 ~ 0.17	0.87
Jin et al/2021 [16]	/	/	/	0	0.43	WMD = - 0.005	- 0.17 ~ - 0.16	0.95
Zhang Z et al/2021 [17]	5	343	655	66	0.02	WMD = - 0.24	- 0.68 ~ 0.19	0.27
Feng et al/2021 [19]	6	1117	1319	0	0.45	WMD = - 0.02	- 0.20 ~ 0.17	0.85
Gong et al/2022 [20]	6	1176	1614	37	0.18	WMD = - 0.08	- 0.42 ~ 0.25	0.63

Table S3. Distal margin length.

Authors/Year	No. of studies	Sample size		Heterogeneity		Overall effect size	95% CI of overall effect	P value
		RG	LG	I^2 (%)	P value			
Ma et al/2020 [14]	6	710	1194	59	0.03	WMD = - 0.15	- 0.52 ~ 0.21	0.41
Guerrini et al/2020 [15]	12	1889	4920	79	<0.00001	WMD = 0.27	-0.15 ~ 0.69	0.21
Jin et al/2021 [16]	/	/	/	52	0.078	WMD = - 0.59	- 0.98 ~ - 0.20	**0.003**
Zhang Z et al/2021 [17]	5	343	655	0	0.46	WMD = 0.21	0.02 ~ 0.40	**0.03**
Feng et al/2021 [19]	6	1117	1319	12	0.34	WMD = 0.22	- 0.13 ~ 0.27	0.51
Gong et al/2022 [20]	6	1176	1614	66	0.01	WMD = - 0.22	- 0.04 ~ 0.48	0.10

Table S4. Mortality.

Authors/Year	No. of studies	Sample size		Heterogeneity		Overall effect size	95% CI of overall effect	P value
		RG	LG	I^2 (%)	P value			
Ma et al/2020 [14]	5	762	1386	0	0.82	OR = 0.67	0.24 ~ 1.90	0.45
Guerrini et al/2020 [15]	30	4378	10354	0	0.95	OR = 1.43	0.77 ~ 2.65	0.25
Jin et al/2021 [16]	7	/	/	0	0.93	OR = 1.28	0.51 ~ 3.21	0.59
Zhang Z et al/2021 [17]	6	520	1032	0	0.92	OR = 0.69	0.37 ~ 1.27	0.23
Zhang X et al/2021 [18]	5	669	858	0	0.79	RR = 1.25	0.49 ~ 3.22	0.64
Gong et al/2022 [20]	7	1233	1741	0	0.61	OR = 1.60	0.60 ~ 4.29	0.35

Table S5. Reoperation rate.

Authors/Year	No. of studies	Sample size		Heterogeneity		Overall effect size	95% CI of overall effect	P value
		RG	LG	I^2 (%)	P value			
Guerrini et al/2020 [15]	15	1939	4467	0	0.60	OR = 1.01	0.60 ~ 1.68	0.98
Jin et al/2021 [16]	8	/	/	0	0.92	OR = 0.85	0.48 ~ 1.52	0.60
Feng et al/2021 [19]	5	1197	1464	0	0.95	OR = 0.63	0.33 ~ 1.20	0.16
Gong et al/2022 [20]	4	482	788	0	/	OR = 0.78	0.30 ~ 2.06	0.62

Table S6. Anastomotic leakage.

Authors/Year	No. of studies	Sample size		Heterogeneity		Overall effect size	95% CI of overall effect	P value
		RG	LG	I^2 (%)	P value			
Guerrini et al/2020 [15]	26	4045	9595	0	0.89	OR = 1.04	0.79 ~ 1.37	0.78
Jin et al/2021 [16]	/	/	/	0	0.93	OR = 0.88	0.60 ~ 1.30	0.53
Zhang Z et al/2021 [17]	9	885	1517	0	0.92	OR = 0.84	0.45 ~ 1.58	0.59
Zhang X et al/2021 [18]	7	927	1100	0	0.94	RR = 0.58	0.30 ~ 1.10	0.10
Feng et al/2021 [19]	14	4474	4943	0	0.67	OR = 0.93	0.67 ~ 1.29	0.67
Gong et al/2022 [20]	7	1073	1708	0	/	OR = 0.88	0.36 ~ 2.15	0.78

Table S7. Delayed gastric emptying.

Authors/Year	No. of studies	Sample size		Heterogeneity		Overall effect size	95% CI of overall effect	P value
		RG	LG	I^2 (%)	P value			
Jin et al/2021 [16]	/	/	/	0	0.91	OR = 1.16	0.56 ~ 2.41	0.69
Zhang Z et al/2021 [17]	6	447	434	0	0.90	OR = 1.44	0.62 ~ 3.34	0.39
Zhang X et al/2021 [18]	5	590	750	0	0.87	RR = 1.14	0.47 ~ 2.75	0.78

Table S8. Intestinal obstruction.

Authors/Year	No. of studies	Sample size		Heterogeneity		Overall effect size	95% CI of overall effect	P value
		RG	LG	I^2 (%)	P value			
Jin et al/2021 [16]	/	/	/	0	0.65	OR = 1.05	0.48 ~ 2.31	0.90
Zhang X et al/2021 [18]	5	812	947	0	0.66	RR = 0.70	0.27 ~ 1.87	0.48
Gong et al/2022 [20]	6	783	1046	0	/	OR = 0.95	0.38 ~ 2.34	0.91

Table S9. Wound infection.

Authors/Year	No. of studies	Sample size		Heterogeneity		Overall effect size	95% CI of overall effect	P value
		RG	LG	I^2 (%)	P value			
Jin et al/2021 [16]	/	/	/	0	0.84	OR = 0.94	0.62 ~ 1.44	0.79
Zhang X et al/2021 [18]	7	927	1100	0	0.95	RR = 0.74	0.42 ~ 1.30	0.29

Table S10. Intra-abdominal infection.

Authors/Year	No. of studies	Sample size		Heterogeneity		Overall effect size	95% CI of overall effect	P value
		RG	LG	I^2 (%)	P value			
Zhang X et al/2021 [18]	5	748	876	0	0.84	RR = 0.76	0.29 ~ 2.02	0.58
Gong et al/2022 [20]	6	582	1305	0	/	OR = 0.88	0.35 ~ 2.25	0.80

Table S11. Duodenal stump leakage.

Authors/Year	No. of studies	Sample size		Heterogeneity		Overall effect size	95% CI of overall effect	P value
		RG	LG	I^2 (%)	P value			
Feng et al/2021 [19]	6	3192	3458	0	0.69	OR = 0.88	0.53 ~ 1.45	0.61
Gong et al/2022 [20]	4	940	1002	0	/	OR = 0.96	0.43 ~ 2.11	0.91

Table S12. Anastomotic stenosis.

Authors/Year	No. of studies	Sample size		Heterogeneity		Overall effect size	95% CI of overall effect	P value
		RG	LG	I^2 (%)	P value			
Feng et al/2021 [19]	5	2630	2630	0	0.57	OR = 1.00	0.48 ~ 2.08	1.00

Table S13. Abdominal bleeding.

Authors/Year	No. of studies	Sample size		Heterogeneity		Overall effect size	95% CI of overall effect	P value
		RG	LG	I^2 (%)	P value			
Feng et al/2021 [19]	12	4001	4470	0	0.83	OR = 0.66	0.41 ~ 1.07	0.09

Table S14. Ileus.

Authors/Year	No. of studies	Sample size		Heterogeneity		Overall effect size	95% CI of overall effect	P value
		RG	LG	I^2 (%)	P value			
Feng et al/2021 [19]	12	4424	4893	0	0.74	OR = 0.82	0.52 ~ 1.28	0.38

Table S15. Pneumonia.

Authors/Year	No. of studies	Sample size		Heterogeneity		Overall effect size	95% CI of overall effect	P value
		RG	LG	I^2 (%)	P value			
Zhang X et al/2021 [18]	6	877	1044	0	0.87	RR = 0.84	0.41 ~ 1.74	0.65

Table S16. Overall survival.

Authors/Year	No. of studies	Sample size		Heterogeneity		Overall effect size	95% CI of overall effect	P value
		RG	LG	I^2 (%)	P value			
Ma et al/2020 [14]	6	890	1498	0	0.86	HR = 0.95	0.76 ~ 1.18	0.64
Jin et al/2021 (3 yr) [16]	5	/	/	1	0.40	OR = 1.03	0.78 ~ 1.35	0.83
Jin et al/2021 (5 yr) [16]	3	/	/	68	0.04	OR = 0.86	0.72 ~ 1.03	0.10
Feng et al/2021 [19]	7	3475	4106	0	0.80	HR = 0.96	0.86 ~ 1.07	0.50

Table S17. Recurrence free-survival.

Authors/Year	No. of studies	Sample size		Heterogeneity		Overall effect size	95% CI of overall effect	P value
		RG	LG	I^2 (%)	P value			
Ma et al/2020 [14]	3	586	586	0	0.91	HR = 0.91	0.69 ~ 1.21	0.53
Feng et al/2021 [19]	5	2732	2732	0	0.68	HR = 0.98	0.80 ~ 1.21	0.85

Table S18. Recurrence.

Authors/Year	No. of studies	Sample size		Heterogeneity		Overall effect size	95% CI of overall effect	P value
		RG	LG	I^2 (%)	P value			
Ma et al/2020 [14]	5	757	1038	0	0.62	OR = 0.90	0.67 ~ 1.21	0.50
Guerrini et al/2020 [15]	10	1322	1942	0	0.97	OR = 0.86	0.67 ~ 1.11	0.25
Zhang Z et al/2021 [17]	3	231	222	0	0.91	OR = 0.69	0.39 ~ 1.23	0.21
